# Incarceration history and HIV testing among people who inject drugs in the Boston metro area: a pooled cross-sectional study

**DOI:** 10.21203/rs.3.rs-5367945/v1

**Published:** 2024-12-17

**Authors:** Benjamin J. Bovell-Ammon, Shauna Onofrey, Simeon D. Kimmel, Alysse G. Wurcel, Monina Klevens

**Affiliations:** UMass Chan Medical School-Baystate and Baystate Medical Center; Massachusetts Department of Public Health; Boston Medical Center and Boston University Chobanian and Avedisian School of Medicine; Boston Medical Center and Boston University Chobanian and Avedisian School of Medicine; Harvard University

**Keywords:** People who inject drugs, HIV testing, incarceration, jail, prison, opioid use disorder

## Abstract

**Background:**

The persistent incidence of HIV among people who inject drugs (PWID) underscores the urgency for HIV prevention efforts to end the HIV epidemic. Little is known about the role carceral settings play as touchpoints for HIV testing in this population.

**Methods:**

Secondary analysis of cross-sectional survey data of PWID in the Boston metro area from the 2015 and 2018 cycles of the National HIV Behavioral Surveillance (NHBS). Among self-reported HIV-negative participants, we examined incarceration and HIV testing histories and used a multivariable modified Poisson regression model to evaluate the association between incarceration history (main exposure) and past-year HIV testing (primary outcome).

**Results:**

Among 957 participants, average age was 38.9 (SD 11.1) years, 70.1% were male, 15.2% were Hispanic (of any race), 8.4% were non-Hispanic Black, and 68.1% were non-Hispanic White. Regarding incarceration experiences, 43.5% of participants reported past-year incarceration, and 41.8% reported a history of incarceration but only prior to the past year. Among those with past-year incarceration, 23.4% said their last HIV test was done at a jail or prison. Adjusting for other characteristics, compared to no incarceration history, past-year incarceration (PR 1.39; 95% CI: 1.29, 1.49) and incarceration prior to the the past year (PR 1.19; 95%CI: 1.02, 1.38) were both associated with a greater prevalence of past-year HIV testing.

**Conclusions:**

Among PWID, incarceration was very common and was a substantial source of HIV testing. However, more testing is still needed—in both community and carceral settings—to reach optimal testing rates in this key population.

## Introduction

While the overall incidence of HIV in the United States (US) has decreased since 2015, the HIV incidence attributed to injection drug use is stable nationally (1,2) and even increasing in some places (3,4). Alongside the rise in opioid use and use disorders in the US, the prevalence of injection drug use has also increased over the past decade to an estimated 3,700,000 (or 1.5% of US adults) in 2018 (2,5–7). Moreover, the increasing presence of fentanyl in illicit drug markets has intensified the risks of infectious complications faced by PWID who inject opioids (in addition to precipitating an unprecedented surge in overdose deaths since 2013) (8,9). Because of its shorter pharmacologic half-life, fentanyl is injected more frequently and may be associated with greater risk of sharing injection equipment (10,11). Several HIV outbreaks have occurred among people who inject drugs (PWID) (12–15), often associated with state, federal or local policies preventing access to harm reduction tools.

Considering their high risk for infection, HIV testing rates among PWID are often inadequate (16,17). HIV testing is a core pillar of the US Department of Health and Human Services’ Ending the HIV Epidemic campaign (18). The US Centers for Disease Control and Prevention (CDC) recommends that PWID get HIV testing at least annually (more often depending on risk behaviors) (19), but in 2018, for example, only 57% of a national sample of PWID from metropolitan areas reported HIV testing within the past year (20). HIV testing is an opportunity to diagnose the infection, educate about risk reduction, and deliver evidence-based prevention strategies such as pre-exposure prophylaxis, which is underutilized among PWID (21). PWID face substance use, stigma, high rates of homelessness, and barriers to care—which shape the risk environment for HIV and access to harm reduction services (22–29).

Due to the criminalization of substance use, contact with the criminal-legal system, such as incarceration, continues to be highly prevalent among PWID (30), with one third of a national sample of PWID reporting past-year incarceration in 2018 (16). A growing literature describes incarceration as a key social-structural determinant of health and health care (31–35), but its relationship with HIV testing utilization in this population has not been well-described. The CDC recommends that all carceral facilities provide voluntary, opt-out HIV testing (19,36), but many facilities in the US do not provide this, especially jails and less urban jurisdictions (37–40). On one hand, therefore, incarceration might be a point of contact for some PWID that increases access to testing (and other health services) (41). On the other hand, incarceration directly disrupts engagement in health services, can lead to increased risk behavior after release, and deteriorates the resources (e.g. income, housing, social support) that are needed to facilitate access to care in the community (42–49). Although testing should occur in carceral settings, the overlay of stigma, mistrust, lack of resources, low prioritization, and disjointed healthcare systems may prevent access to HIV testing in jails and prisons.

With a statewide opioid use disorder (OUD) prevalence of approximately 5% (50), Massachusetts has high rates of opioid-related morbidity and mortality (9,51–54) as well as increasing incidence of HIV attributable to injection drug use (5% in 2014 to 14% in 2020) (3,4). HIV outbreaks in this population in the northeastern part of the state (10,12,55) and in Boston highlight the need for improved HIV diagnosis and prevention among PWID (56–58). Therefore, Massachusetts is a suitable setting to examine HIV testing among PWID and the association of incarceration history with HIV testing.

## Methods

### Data Source and Sample Selection

We conducted a secondary analysis of a pooled dataset of cross-sectional studies conducted in the Boston area as part of the 2015 and 2018 PWID cycles of the National HIV Behavioral Surveillance (NHBS) system. A multisite project funded by the CDC, NHBS conducts rotating annual cycles of biobehavioral data collection from three specific populations with a high burden of HIV (people who inject drugs, men who have sex with men, and people with high-risk heterosexual behavior) in multiple major cities across the US (20 cities in 2015 and 23 cities in 2018). During PWID cycles, NHBS recruited eligible individuals in the community to participate in an interviewer-administered risk behavior survey and to take an HIV test. NHBS used a respondent-driven sampling (RDS) design, where researchers first recruit a limited number of ‘seed’ participants and then incentivize participants to recruit additional participants through their existing social networks (who in turn can recruit others as well), leading to distinct recruitment ‘chains,’ or clusters (59–61). Eligibility criteria included being 18 years of age or older, a history of injection drug use within the past 12 months, and ability to complete the survey in either English or Spanish. Participation was anonymous and voluntary. NHBS obtained verbal informed consent before conducting the survey and the HIV test and offered financial incentives for completion of the survey, HIV test, and recruitment of additional participants, respectively. NHBS informed participants of their test results and (if indicated) referred them to treatment or other services, while maintaining their anonymity with respect to NHBS participation (62,63). At the Boston site, which was administered by the Massachusetts Department of Public Health (MADPH) with the support of the CDC, NHBS recruited PWID from a five-county sampling area that covered the Boston metropolitan area.

For this secondary analysis of Boston-area NHBS data, we defined the study sample as those who were eligible for HIV testing within the 12 months prior to NHBS participation, which included the following: 1) participants who reported negative or unknown HIV status at the time of participation and 2) those who reported that they had first been diagnosed with HIV within the preceding 12 months (because these individuals were also eligible for HIV testing at some point within the past 12 months). In other words, we only excluded HIV-positive participants from our sample if they reported being diagnosed with HIV more than 12 months prior to participation.

This study was approved by the Institutional Review Board of the MADPH. Reporting in this study followed applicable Strengthening the Reporting of Observational Studies in Epidemiology (STROBE) guidelines for cross-sectional studies.

### Measures

The main exposure of interest was incarceration history. Two linked NHBS questions gathered data about incarceration history: (1) “Have you ever been held in a detention center, jail, or prison for more than 24 hours?” and, if so, they were then asked (2) whether this had occurred during the past 12 months. Notably, the survey questions did not differentiate between detention (i.e. brief jail stay while awaiting law enforcement or judicial proceedings) and incarceration (i.e. custodial sentence for a crime, served in a jail or prison), yet in this study we use the term “incarceration” to refer to any affirmative response to these survey questions. Based on these two questions, we categorized participants into three mutually exclusive levels of incarceration history: past-year incarceration (any history of incarceration within the past 12 months), incarceration prior to the past 12 months, and no incarceration history.

The primary outcome was self-reported HIV testing in the past 12 months (hereafter, past-year testing), which we analyzed using a regression analysis (described below). To complement the regression analysis, we used additional survey items for descriptive analyses of other aspects of participants’ testing histories, including the following: whether they had received an HIV test while incarcerated in the past year; total number of HIV tests in the past 2 years; whether they had used a rapid home HIV test in the past year, past-year testing for bacterial sexually transmitted infections (STIs; i.e. gonorrhea, chlamydia, or syphilis); the location of their most recent HIV test; and, if no HIV test within the past year, the most important reason for not testing.

### Statistical Analysis

We used cross-tabulations to compare participant characteristics and various aspects of HIV testing history (HIV testing rates; and the location of last HIV test or the reasons for not getting tested, as applicable) by incarceration history. We analyzed the association between the primary outcome, past-year HIV testing, and the main exposure, incarceration history, using modified Poisson regression models which accounted for the clustering of observations resulting from the RDS sampling design, i.e. correlations among participants within each recruitment chain (64,65). Our multivariable regression model adjusted for various self-reported demographic, social, behavioral, and clinical characteristics that we hypothesized *a priori* are related to risk of HIV acquisition or access to HIV testing: age, gender, race and ethnicity (provided by NHBS as a joint variable: Hispanic/Latino of any race, non-Hispanic Black, non-Hispanic White, or other), marital status, education, income, homelessness, sexual activity, injection frequency, receptive sharing of injection equipment (i.e. needles, cooker, cotton, or water), stimulant injection, binge drinking, drug treatment program participation, syringe service program utilization, usual source of medical care, NHBS round (2015 vs. 2018), and size of PWID social network (because of its relevance to RDS design effects). For the self-reported gender variable above, NHBS asked participants “Do you consider yourself to be male, female, or transgender?” and we used these three gender categories. We retained the transgender participants in the sample for all descriptive analyses, but we excluded them from the regression analysis due to small numbers (n=6) that would not have allowed valid statistical inference. We also excluded participants from the regression analysis if they had missing values at one or more of the model variables (variables with missingness are indicated in Table 1). Taken together, these two criteria (transgender and missingness) excluded a total of 19 participants (2.0% of our total study sample) from the regression analysis. We included some additional descriptive variables in Table 1 which we did not use as covariates in the regression analysis for the sake of parsimony, avoiding collinearity, or other reasons: namely, employment (model already included income, education and homelessness as markers of socioeconomic status), insurance coverage (lack of heterogeneity and redundant as a marker of socioeconomic status), non-injection drug use (a priori not a predictor of HIV testing), and prior HCV diagnosis (overly correlated with HIV testing). We used SAS, version 9.4 (SAS Institute Inc.) for all analyses. Two-sided P < 0.05 indicated statistical significance.

## Results

Of the 957 participants included in our study sample, the average age was 38.9 (SD 11.1) years, 671 (70.1%) were male, 145 (15.2%) were Hispanic (of any race), 80 (8.4%) were non-Hispanic Black, and 651 (68.1%) were non-Hispanic White. Most participants had a history of incarceration at some point, with 416 (43.5%) reporting past-year incarceration and 400 (41.8%) reporting only a history of incarceration prior to the past year, while the remaining 141 (14.7%) had never been incarcerated (Table 1). Participant characteristics were similar between those with incarceration in the past year and prior to the past year, except that those with a less recent incarceration history were older on average and less likely to report recent binge drinking. Compared to the two groups with incarceration histories, those without any history of incarceration were more likely to be female and have a higher education level and less likely to report an HCV diagnosis.

Overall, 93.8% of participants reported ever being tested for HIV, and 58.5% reported past-year HIV testing (Table 2). Those with past-year incarceration were more likely to report past-year HIV testing (74%) compared to the other two groups (incarcerated prior to past year, 62%; never incarcerated, 62%). Among those with past-year incarceration, 30.5% reported receiving an HIV test while incarcerated. Self-testing with rapid HIV tests was rare (1.4% overall). Compared to past-year HIV testing, past-year testing for bacterial STIs was lower overall (45.1%) and in each group, and those with past-year incarceration were slightly more likely than those in the other two groups to report STI testing.

**Figure 1:**
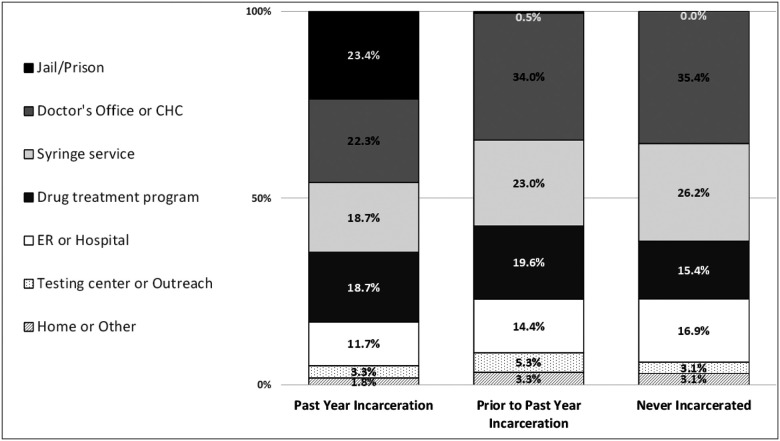
Location of Last HIV Test, by Incarceration History Self-reported locations of participants’ last HIV tests, by incarceration history. Due to incomplete response rates, the number of responses among those with past year incarceration was n=273; among those with incarceration prior to the past year, n=209; and for those with no incarceration history, n=65. All values represent column percentages (column sums may be greater than 100% due to rounding).

Locations of most recent HIV test varied across the three categories of groups (Figure 1). Among those with past-year incarceration, ‘correctional facility’ was tied with ‘doctor’s office or community health center’ as their most common response.

Among those without past-year HIV testing, the main reason reported for not getting tested in the past 12 months varied across the incarceration groups (Table 3). Among those with past-year incarceration and those with no incarceration history, the most important reason was ‘You were afraid of finding out that you have HIV.’ Among those with incarceration prior to the past year, that response was as common as, ‘You think you are at low risk for HIV infection.’ ‘No particular reason’ was common among all groups, chosen by more than a third in each group.

In the multivariable regression analysis (Table 4), those with past-year incarceration had significantly greater prevalence of past-year HIV testing compared to those with no incarceration history (adjusted prevalence ratio [aPR] 1.39; 95% CI: 1.29, 1.49), and, to a lesser extent, so did those with incarceration prior to the past year (aPR 1.19; 95%CI: 1.02, 1.38). Other statistically significant factors associated with greater prevalence of past-year HIV testing in the multivariable-adjusted analysis were younger age, homelessness, only heterosexual activity (vs. any male-to-male), not binge drinking, drug treatment program participation, syringe service program participation, and having a usual source of medical care.

## Discussion

In this secondary analysis of survey data from PWID in the Boston metro area, 58.5% of participants reported receiving an HIV test within the past 12 months, which is similar to the overall testing rate of the national NHBS sample (57–58% in 2015 and 2018) (66,67). However, the goal among PWID should be nearly universal coverage, in keeping with the CDC recommendations to test at least annually (19). Having a history of incarceration, whether it was within the past year or more remote, was significantly associated with HIV testing, which underscores the role that incarceration currently plays in HIV testing for a vulnerable group at high risk for HIV. Among PWID with past-year incarceration, approximately three quarters had received an HIV test in the past year, with three out of ten receiving that test while incarcerated.

This association between incarceration and HIV testing, which has also been seen elsewhere among PWID (68) and other groups in the US (69–72), highlights several challenges in delivering HIV testing to PWID—the fact that substance use and injection drug use are criminalized concentrates this high risk population in jails and prisons. Given the deleterious effects that incarceration can have on health and healthcare, both in terms of HIV and otherwise (31–35,48,73,74), we are not suggesting that incarceration is a beneficial solution to problems of healthcare access in the community. In fact, we argue that the overlapping developments with HIV, injection drug use, and mass incarceration over the past two decades in the US represent a syndemic (75), a synergistic interaction between multiple health conditions and social vulnerabilities within a specific population (30,73,76,77). Nevertheless, we also maintain that public health efforts should take advantage of existing touchpoints to offer HIV testing (and other prevention and treatment measures) to PWID (41).

Like in the community, there are still missed opportunities to engage PWID in testing in carceral settings. Indeed, we still found that over one third of recently incarcerated individuals had no past-year HIV testing. There are several reasons why participants may not have been tested in carceral settings. In Massachusetts, verbal consent is sufficient for HIV testing (Massachusetts General Laws Ch. 111, Section 70F) (78), yet most jails in the state require written consent. A 2018 study of testing policies across Massachusetts county jails found that none of the jails offered universal opt-out HIV testing at intake, and less than half (46%) of them routinely offered opt-in testing (40). Some facilities might not offer testing because of the cost of providing medications for identified cases, and thus some may not know that the US government’s Ryan White HIV/AIDS Program can cover HIV treatment in jails and prisons (79). While CDC guidelines recommend universal opt-out testing in carceral facilities (19,36), some have raised questions about the best way to offer testing. While one study found better uptake when opt-out testing was offered on the day of admission (compared to the next day or to one week later) (80), another study found better uptake when opt-out testing was integrated with routine phlebotomy and separate from the often chaotic intake process (81). With the concerns about coercion, confidentiality, and stigma during incarceration (82) and the resulting mistrust that many feel, one study of incarcerated individuals’ perspectives found that most participants preferred the opt-in approach (over the opt-out approach) because it gave them a greater sense of autonomous choice (83). In terms of the most suitable testing modality to use in carceral settings, one study found, perhaps counterintuitively, that uptake (and the total number who both were tested and received their result) improved when a large urban jail switched from a rapid point-of-care test to a laboratory-based test that required phlebotomy (84); yet whether this finding holds true among PWID specifically, who often have more difficulties with phlebotomy (85), may warrant further investigation. One other advantage of laboratory-based HIV testing is the relative ease of combining it with other tests at the same time, such as hepatitis C virus, tuberculosis, or syphilis (81,86).

Among people who received HIV testing, a large percentage of tests occurred outside of mainstream medical settings (despite the fact that over 80% of participants reported having a usual source of medical care). In our sample, HIV testing happened approximately just as often in syringe service programs and drug treatment programs as it did in doctor’s offices/community health centers, emergency departments and carceral settings. These patterns of HIV testing sites signal that high-impact testing programs should be offered in locations that PWID already frequent (41,87,88). Efforts are needed to strengthen HIV testing services in substance use treatment facilities, which would require, among other things, public health investment and reforms in payment for substance use treatment. Scholars have also noted the importance of on-demand rapid testing for PWID, a modality that could facilitate HIV testing outside of mainstream healthcare settings and which avoids some of the structural barriers and stigma associated with laboratory-based tests that require phlebotomy and subsequent follow-up for results (85). Future research could explore why homelessness, which was reported by approximately two thirds of respondents, was associated with a greater prevalence of HIV testing—our multivariable regression model adjusted for syringe service program use and incarceration so some other factor(s) may explain this. We also found that bacterial STI testing was much lower than HIV testing in this PWID sample, suggesting that the elevated sexual transmission risk in this population, which is already known, may be relatively overlooked (89). Use of rapid self tests was rare in our sample, which may represent an opportunity.

Our study has limitations. The cross-sectional data source prohibits causal inference, and the summary measures of past-year incarceration and past-year testing did not allow us to identify the relative order in which these had occurred. We also lacked other information about incarceration, e.g. brief jail detention vs. long imprisonment, reason for incarceration. Self-reported information about stigmatized behaviors, such as substance use and sexual activity, could have been susceptible to social desirability bias. For location of testing, our data only described the last test location for each individual, which might not be representative of the total amount of testing being utilized from each location. Our findings from a single site, the Boston metro area, might not generalize to other places, as carceral systems and policies and harm reduction services for PWID can vary widely across the US. Also, respondent-driven sampling might not be representative of the entire PWID population.

## Conclusion

In this cross-sectional study of Boston-area PWID, contact with carceral facilities was very common and was also a substantial source of HIV testing. However, overall rates of testing need to be improved, which would require more testing in both community and carceral facilities. There should be “no wrong door” for accessing testing, i.e. HIV testing should be available wherever possible for PWID, taking advantage of existing touchpoints.

## Figures and Tables

**Table 1 –:** Sample Characteristics (N=957)

		Incarceration History		
	Past year	Prior to past year	Never	p-value
	(n=416)	(n=400)	(n=141)	
Survey Year				
2015	58.9	47.5	49.7	
2018	41.1	52.5	50.4	
Age, mean (SD)	36.4 (9.6)	42.7 (11.4)	36.0 (11.2)	<.0001
Gender^a^				<.0001
Female (cis-gender)	23.8	26.5	53.2	
Male (cis-gender)	76.0	72.5	46.1	
Transgender	0.2	1.0	0.7	
Race/ethnicity				.17
White, non-Hispanic	69.4	65.0	73.1	
Black, non-Hispanic	7.0	11.0	5.0	
Hispanic (any race)	15.9	15.5	12.1	
Other non-Hispanic *(missing n=1)*	7.7	8.5	9.9	
Marital status				<.01
Married or cohabitating	8.2	12.3	12.8	
Divorced, separated, or widowed	20.9	29.5	26.2	
Single	70.9	58.3	61.0	
High school completion *(missing n=1)*	77.6	73.0	84.3	.02
Homelessness, current *(missing n=1)*	71.6	61.5	58.2	<.01
Employment, current	13.7	12.3	17.0	.36
Income below federal poverty level, past year *(missing n=4)*	72.3	74.2	67.6	.33
Inject more than once per day, past year *(missing n=1)*	76.2	68.9	66.7	.02
Receptive sharing of injection equipment, past year	82.7	74.3	76.6	.01
Opioid injection, past year	96.9	95.5	97.2	.49
Stimulant injection, past year	78.6	68.3	68.1	<.01
Non-injection drug use, past year	87.5	86.3	86.5	.86
Binge drinking, past 30 days *(missing n=9)*	42.9	33.8	28.1	<.01
Sexual activity, past year				<.01
Any male-to-male	14.4	16.8	9.2	
Heterosexual only	80.8	72.5	83.7	
None	4.8	10.8	7.1	
Transactional sex, past year *(missing n=6)*	32.5	28.5	24.3	.15
Health insurance, current				.22
No Insurance	3.6	4.5	7.8	
Private, multiple, or other	2.4	2.8	4.3	
Public	94.0	92.8	87.9	
Has a usual source of medical care	85.6	88.3	82.3	.18
Syringe service program utilization, past year	82.5	80.3	76.6	.30
Drug treatment program participation, past year	76.7	67.5	61.7	<.001
Hepatitis C virus infection, self-reported *(missing n=77)*	75.1	74.8	55.3	<.0001

**Table 2 –:** HIV and Bacterial STI Testing History by Incarceration History

	Overall	Incarceration History		
		Past Year	Prior to Past Year	Never
	*(n=957)*	*(n=416)*	*(n=400)*	*(n=141)*
HIV Testing, Ever (Lifetime)	93.8	94.5	94.5	90.1
HIV Testing, Past Year	58.5	67.0	53.4	47.9
HIV Testing While Incarcerated, Past Year	n/a	30.5	n/a	n/a
Total No. HIV Tests in Past 2 Years, median (IQR)	2 (1,3)	2 (1,3)	2 (1,3)	1 (0,3)
Rapid Home HIV Test, Past Year	1.4	1.9	1.0	0.7
Bacterial STI Testing, Past Year	45.1	50.1	40.8	42.1
Bacterial STI Diagnosis, Past Year	5.0	5.1	4.8	5.7

All values in table are column percentages unless otherwise specified.

a Gender categories reflect participants’ responses to the following survey question: “Do you consider yourself to be male, female or transgender?”

Note: All values represent column percentages unless otherwise specified. Abbreviations: STI, sextually transmitted infection; IQR, interquartile range.

**Table 3 -:** Reasons for Not Receiving Past-Year HIV Test, by Incarceration History

	Past Year Incarceration	Prior to Past Year Incarceration	Never incarcerated
	*(n=137)*	*(n=185)*	*(n=73)*
You think you are at low risk for HIV infection?	12.4	24.3	16.4
You were afraid of finding out that you had HIV?	41.6	27.0	30.1
You didn’t have time?	8.8	6.5	6.9
Some other reason?	2.9	3.8	5.5
No particular reason	34.3	38.4	41.1

Among participants that had not received HIV testing within the past year, this table records their responses to the following survey question: “Which of these best describes the most important reason you have not been tested for HIV in the past 12 months?” All values represent column percentages (column sums may be greater than 100% due to rounding).

**Table 4 –:** Associations with Past-Year HIV Testing Using Modified Poisson Regression (N=938)^a^

	Unadjusted				Adjusted (Multivariable)			
	PR		95% CI		aPR		95% CI	
Incarceration History								
Past year	1.40	*	1.26	0.54	1.39	*	1.29	1.49
Prior to past year	1.12	*	0.96	1.29	1.19	*	1.02	1.38
Never	(ref.)				(ref.)			
Age (in years)	0.99		0.99	0.99	0.99	*	0.99	1.00
Male gender (vs. Female)^b^	0.99		0.91	1.08	1.02		0.94	1.12
Race/Ethnicity								
White, non-Hispanic	(ref.)				(ref.)			
Black, non-Hispanic	0.88		0.74	1.04	1.10		0.85	1.42
Hispanic of any race	0.88		0.66	1.19	0.90		0.71	1.15
Other non-Hispanic	0.94		0.82	1.08	1.03		0.88	1.20
Education, completed HS (vs. did not complete HS)	1.13		0.88	1.45	1.16		0.95	1.41
Income at or below FPL (vs. above FPL)	1.05		0.94	1.17	1.06		0.96	1.16
Marital status								
Single	(ref.)							
Divorced, separated, or widowed	0.85		0.72	1.02	0.95		0.78	1.15
Married or cohabitating	0.96		0.85	1.08	1.05		0.96	1.15
Currently Homeless	1.24	*	1.11	1.38	1.21	*	1.08	1.36
Sexual activity								
Any male-to-male	0.83	*	0.73	0.94	0.85	*	0.79	0.92
Heterosexual only	(ref.)				(ref.)			
None	0.93		0.77	1.12	0.96		0.82	1.12
Inject more than once per day (vs. once per day or less often)	1.00		0.90	1.12	0.97		0.89	1.05
Receptive sharing of injection equipment, past year	1.01		0.90	1.13	0.93		0.86	1.01
Stimulant injection, past year	1.16	*	1.05	1.27	1.08		0.96	1.22
Binge drinking, past 30 days	0.94		0.84	1.04	0.90	*	0.82	0.99
Drug treatment program participation, past year	1.32	*	1.15	1.52	1.25	*	1.12	1.40
Syringe service program utilization, past year	1.28	*	1.11	1.47	1.23	*	1.11	1.38
Has a usual source of medical care	1.10		0.97	1.25	1.14	*	1.02	1.27
Survey Year 2015 (vs. 2018)	1.05		0.94	1.16	1.00		0.94	1.06

Abbreviations: PR, prevalence ratio; aPR, adjusted prevalence ratio; HS, high school; FPL, federal poverty level.

* p<0.05

a The regression model excluded n=19 participants because of missing responses to the model variables and/or because the transgender subgroup was too small to include, as described in the text.

b We excluded transgender individuals from this regression analysis due to very small numbers. Gender categories (female, male, transgender) were based on responses to the following survey question: “Do you consider yourself to be male, female or transgender?”

## Data Availability

The data analyzed in this study (National HIV Behavioral Surveillance data collected at the Boston site) are restricted and managed by the Massachusetts Department of Public Health. Inquiries about the Boston area NHBS data should be directed to shauna.onofrey@mass.gov. The code used in this analysis is available from the authors upon reasonable request.

## Abbreviations

HIV: human immunodeficiency virus
PWID: people who inject drugs
NHBS: National HIV Behavioral Surveillance
US: United States (of America)
CDC: Centers for Disease Control and Prevention (US)
OUD: opioid use disorder
RDS: respondent-driven sampling
MADPH: Massachusetts Department of Public Health
STI: sexually transmitted infection
HCV: hepatitis C virus

## References

[1] Centers for Disease Control and Prevention. Estimated HIV incidence and prevalence in the United States, 2015–2019. HIV Surveillance Supplemental Report [Internet]. 2021 May [cited 2024 Feb 27];26(1). Available from: https://www.cdc.gov/hiv/library/reports/hiv-surveillance.html

[2] Centers for Disease Control and Prevention. Estimated HIV incidence and prevalence in the United States, 2017–2021. HIV Surveillance Supplemental Report [Internet]. 2023 May [cited 2024 Feb 27];28(3). Available from: https://stacks.cdc.gov/view/cdc/149080

[3] Brown CM. MDPH Clinical Advisory: Statewide Outbreak of HIV Infection in Persons who Inject Drugs, February 5, 2019 [Internet]. Massachusetts Department of Public Health; 2019 Feb p. 2. Available from: https://www.mass.gov/files/documents/2019/02/07/statewide%20advisory%20hiv%20in%20pwid%202-5-19.docx?_ga=2.182212583.1590938026.1659456172-1778656474.1644510133

[4] Massachusetts Department of Public Health, Bureau of Infectious Disease and Laboratory Sciences. Massachusetts HIV Epidemiologic Pro le: Data as of 1/1/2022, Population Report: Persons Who Inject Drugs [Internet]. 2023 Apr [cited 2024 Feb 6]. Available from: https://www.mass.gov/lists/hivaids-epidemiologic-profiles

[5] Tempalski B, Pouget ER, Cleland CM, Brady JE, Cooper HLF, Hall HI, Trends in the Population Prevalence of People Who Inject Drugs in US Metropolitan Areas 1992–2007. PLOS ONE [Internet]. 2013 Jun 5 [cited 2022 Jul 18];8(6):e64789. Available from: https://journals.plos.org/plosone/article?id=10.1371/journal.pone.0064789. 23755143 10.1371/journal.pone.0064789 PMC3673953

[6] Lansky A, Finlayson T, Johnson C, Holtzman D, Wejnert C, Mitsch A, Estimating the Number of Persons Who Inject Drugs in the United States by Meta-Analysis to Calculate National Rates of HIV and Hepatitis C Virus Infections. PLOS ONE [Internet]. 2014 May 19 [cited 2022 Jul 19];9(5):e97596. Available from: https://journals.plos.org/plosone/article?id=10.1371/journal.pone.0097596 24840662 10.1371/journal.pone.0097596 PMC4026524

[7] Bradley H, Hall E, Asher A, Furukawa N, Jones CM, Shealey J, Estimated number of people who inject drugs in the United States. Clinical Infectious Diseases [Internet]. 2022 Jul 6 [cited 2022 Jul 19];ciac543. Available from: 10.1093/cid/ciac543 PMC10202436 35791261

[8] Mattson CL. Trends and Geographic Patterns in Drug and Synthetic Opioid Overdose Deaths — United States, 2013–2019. MMWR Morb Mortal Wkly Rep [Internet]. 2021 [cited 2022 Jul 18];70. Available from: https://www.cdc.gov/mmwr/volumes/70/wr/mm7006a4.htm 10.15585/mmwr.mm7006a4 PMC7877587 33571180

[9] O’Donnell JK. Trends in Deaths Involving Heroin and Synthetic Opioids Excluding Methadone, and Law Enforcement Drug Product Reports, by Census Region — United States, 2006–2015. MMWR Morb Mortal Wkly Rep [Internet]. 2017 [cited 2022 Jul 19];66. Available from: https://www.cdc.gov/mmwr/volumes/66/wr/mm6634a2.htm 10.15585/mmwr.mm6634a2 PMC5657786 28859052

[10] Board A, Alpren C, Hernandez B, Murray A, Dawson EL, Drumhiller K, A qualitative study of injection and sexual risk behavior among unstably housed people who inject drugs in the context of an HIV outbreak in Northeast Massachusetts, 2018. International Journal of Drug Policy [Internet]. 2021 Sep 1 [cited 2022 Jul 19];95:103368. Available from: https://www.sciencedirect.com/science/article/pii/S0955395921002735 34390967 10.1016/j.drugpo.2021.103368

[11] Lambdin BH, Bluthenthal RN, Zibbell JE, Wenger L, Simpson K, Kral AH. Associations between Perceived Illicit Fentanyl Use and Infectious Disease Risks among People who Inject Drugs. Int J Drug Policy [Internet]. 2019 Dec [cited 2022 Jul 20];74:299–304. Available from: https://www.ncbi.nlm.nih.gov/pmc/articles/PMC6949008/31733979 10.1016/j.drugpo.2019.10.004 PMC6949008

[12] Alpren C, Dawson EL, John B, Cranston K, Panneer N, Fukuda HD, Opioid Use Fueling HIV Transmission in an Urban Setting: An Outbreak of HIV Infection Among People Who Inject Drugs—Massachusetts, 2015–2018. Am J Public Health [Internet]. 2019 Nov 14 [cited 2021 May 7];110(1):37–44. Available from: https://ajph.aphapublications.org/doi/10.2105/AJPH.2019.305366 31725317 10.2105/AJPH.2019.305366 PMC6893347

[13] Buskin SE, Erly SJ, Glick SN, Lechtenberg RJ, Kerani RP, Herbeck JT, Detection and Response to an HIV Cluster: People Living Homeless and Using Drugs in Seattle, Washington. Am J Prev Med. 2021 Nov;61(5 Suppl 1):S160–9. 34686286 10.1016/j.amepre.2021.04.037

[14] Furukawa NW, Weimer M, Willenburg KS, Kilkenny ME, Atkins AD, Paul McClung R, Expansion of Preexposure Prophylaxis Capacity in Response to an HIV Outbreak Among People Who Inject Drugs-Cabell County, West Virginia, 2019. Public Health Rep. 2022 Feb;137(1):25–31. 33646890 10.1177/0033354921994202 PMC8721767

[15] Peters PJ, Pontones P, Hoover KW, Patel MR, Galang RR, Shields J, HIV Infection Linked to Injection Use of Oxymorphone in Indiana, 2014–2015. N Engl J Med. 2016 Jul 21;375(3):229–39. 27468059 10.1056/NEJMoa1515195

[16] Centers for Disease Control and Prevention. HIV Infection Risk, Prevention, and Testing Behaviors among Persons Who Inject Drugs—National HIV Behavioral Surveillance: Injection Drug Use, 23 U.S. Cities, 2018 [Internet]. 2020 Feb [cited 2021 Jun 16] p. 43. Report No.: 24. Available from: https://www.cdc.gov/hiv/pdf/library/reports/surveillance/cdc-hiv-surveillance-special-report-number-24.pdf

[17] Cooley LA, Wejnert C, Spiller MW, Broz D, Paz-Bailey G, NHBS study Group. Low HIV testing among persons who inject drugs-National HIV Behavioral Surveillance, 20 U.S. cities, 2012. Drug Alcohol Depend. 2016 Aug 1;165:270–4. 27323649 10.1016/j.drugalcdep.2016.05.024 PMC5134421

[18] Fauci AS, Redfield RR, Sigounas G, Weahkee MD, Giroir BP. Ending the HIV Epidemic: A Plan for the United States. JAMA [Internet]. 2019 Mar 5 [cited 2022 Jul 21];321(9):844–5. Available from: 10.1001/jama.2019.1343 30730529

[19] Branson BM, Handsfield HH, Lampe MA, Janssen RS, Taylor AW, Lyss SB, Revised recommendations for HIV testing of adults, adolescents, and pregnant women in health-care settings. MMWR Recomm Rep [Internet]. 2006 Sep 22 [cited 2024 Feb 8];55(RR-14):1–17. Available from: https://www.cdc.gov/mmwr/preview/mmwrhtml/rr5514a1.htm 16988643

[20] Handanagic S, Finlayson T, Burnett JC, Broz D, Wejnert C, National HIV Behavioral Surveillance Study Group. HIV Infection and HIV-Associated Behaviors Among Persons Who Inject Drugs — 23 Metropolitan Statistical Areas, United States, 2018. MMWR Morb Mortal Wkly Rep. 2021 Oct 22;70(42):1459–65. 34673746 10.15585/mmwr.mm7042a1 PMC9361835

[21] Earlywine JJ, Bazzi AR, Biello KB, Klevens RM. High Prevalence of Indications for Pre-exposure Prophylaxis Among People Who Inject Drugs in Boston, Massachusetts. American Journal of Preventive Medicine [Internet]. 2021 Mar [cited 2022 Jul 12];60(3):369–78. Available from: https://linkinghub.elsevier.com/retrieve/pii/S0749379720304451 33229144 10.1016/j.amepre.2020.09.011 PMC7902399

[22] Bazzi AR, Drainoni ML, Biancarelli DL, Hartman JJ, Mimiaga MJ, Mayer KH, Systematic review of HIV treatment adherence research among people who inject drugs in the United States and Canada: evidence to inform pre-exposure prophylaxis (PrEP) adherence interventions. BMC Public Health. 2019 Jan 8;19(1):31. 30621657 10.1186/s12889-018-6314-8 PMC6323713

[23] Earnshaw VA. Stigma and substance use disorders: A clinical, research, and advocacy agenda. American Psychologist [Internet]. 2020 Dec [cited 2022 Feb 28];75(9):1300–11. Available from: https://search.ebscohost.com/login.aspx?direct=true&db=pdh&AN=2020-99903-024&site=ehost-live&scope=site 33382299 10.1037/amp0000744 PMC8168446

[24] Falade-Nwulia O, Sacamano P, McCormick SD, Yang C, Kirk G, Thomas D, Individual and network factors associated with HCV treatment uptake among people who inject drugs. International Journal of Drug Policy [Internet]. 2020 Apr 1 [cited 2021 Oct 12];78:102714. Available from: https://www.sciencedirect.com/science/article/pii/S0955395920300554 32135398 10.1016/j.drugpo.2020.102714 PMC7367433

[25] Flath N, Tobin K, King K, Lee A, Latkin C. Enduring Consequences From the War on Drugs: How Policing Practices Impact HIV Risk Among People Who Inject Drugs in Baltimore City. Subst Use Misuse. 2017 Jul 3;52(8):1003–10. 28318343 10.1080/10826084.2016.1268630 PMC5600621

[26] Hammarlund R, Crapanzano K, Luce L, Mulligan L, Ward K. Review of the effects of self-stigma and perceived social stigma on the treatment-seeking decisions of individuals with drug- and alcohol-use disorders. Subst Abuse Rehabil [Internet]. 2018 Nov 23 [cited 2021 Oct 12];9:115–36. Available from: https://www.ncbi.nlm.nih.gov/pmc/articles/PMC6260179/30538599 10.2147/SAR.S183256 PMC6260179

[27] Meyer JP, Springer SA, Altice FL. Substance Abuse, Violence, and HIV in Women: A Literature Review of the Syndemic. Journal of Women’s Health [Internet]. 2011 Jul [cited 2022 Jul 21];20(7):991–1006. Available from: https://www.liebertpub.com/doi/full/10.1089/jwh.2010.2328 10.1089/jwh.2010.2328 PMC3130513 21668380

[28] Phillips KT. Barriers to practicing risk reduction strategies among people who inject drugs. Addict Res Theory. 2016;24(1):62–8. 27499724 10.3109/16066359.2015.1068301 PMC4972039

[29] Rhodes T. The ‘risk environment’: a framework for understanding and reducing drug-related harm. International Journal of Drug Policy [Internet]. 2002 Jun 1 [cited 2021 May 4];13(2):85–94. Available from: https://www.sciencedirect.com/science/article/pii/S0955395902000075

[30] Zaller N, Brinkley-Rubinstein L. Incarceration, drug use, and infectious diseases: a syndemic still not addressed. The Lancet Infectious Diseases [Internet]. 2018 Dec 1 [cited 2022 Jul 21];18(12):1301–2. Available from: https://www.thelancet.com/journals/laninf/article/PIIS1473-3099(18)30538-3/fulltext 30385159 10.1016/S1473-3099(18)30538-3

[31] Brinkley-Rubinstein L. Incarceration as a catalyst for worsening health. Health & Justice [Internet]. 2013 Oct 24 [cited 2020 Oct 15];1(1):3. Available from: 10.1186/2194-7899-1-3

[32] Brinkley-Rubinstein L, Cloud DH. Mass Incarceration as a Social-Structural Driver of Health Inequities: A Supplement to AJPH. Am J Public Health [Internet]. 2020 Jan 1 [cited 2021 Aug 13];110(S1):S14–5. Available from: https://ajph.aphapublications.org/doi/10.2105/AJPH.2019.305486 31967896 10.2105/AJPH.2019.305486 PMC6987928

[33] Howell BA, Earnshaw VA, Garcia M, Taylor A, Martin K, Fox AD. The Stigma of Criminal Legal Involvement and Health: a Conceptual Framework. J Urban Health [Internet]. 2022 Jan 15 [cited 2022 Jan 31]; Available from: 10.1007/s11524-021-00599-y PMC8866593 35031942

[34] Massoglia M, Pridemore WA. Incarceration and Health. Annu Rev Sociol. 2015 Aug;41:291–310. 30197467 10.1146/annurev-soc-073014-112326 PMC6124689

[35] Wildeman C, Wang EA. Mass incarceration, public health, and widening inequality in the USA. The Lancet [Internet]. 2017 Apr 8 [cited 2019 Sep 29];389(10077):1464–74. Available from: http://www.sciencedirect.com/science/article/pii/S0140673617302593 10.1016/S0140-6736(17)30259-3 28402828

[36] MacGowan RJ. HIV testing implementation guidance for correctional settings. Centers for Disease Control and Prevention, editor. 2009 Jan; Available from: https://stacks.cdc.gov/view/cdc/5279

[37] Maner M, Omori M, Brinkley-Rubinstein L, Beckwith CG, Nowotny K. Infectious disease surveillance in U.S. jails: Findings from a national survey. PLOS ONE [Internet]. 2022 Aug 25 [cited 2022 Aug 28];17(8):e0272374. Available from: https://journals.plos.org/plosone/article?id=10.1371/journal.pone.0272374 36006896 10.1371/journal.pone.0272374 PMC9409583

[38] Maruschak LM, Bronson J. HIV in Prisons, 2015 - Statistical Tables [Internet]. Bureau of Justice Statistics; 2017 Aug [cited 2021 Jun 21] p. 18. (HIV in Prisons and Jails). Report No.: NCJ 250641. Available from: https://bjs.ojp.gov/library/publications/hiv-prisons-2015-statistical-tables

[39] Solomon L, Montague BT, Beckwith CG, Baillargeon J, Costa M, Dumont D, Survey Finds That Many Prisons And Jails Have Room To Improve HIV Testing And Coordination Of Postrelease Treatment. Health Affairs [Internet]. 2014 Mar 1 [cited 2021 Mar 30];33(3):434–42. Available from: https://www.healthaffairs.org/doi/10.1377/hlthaff.2013.1115 24590942 10.1377/hlthaff.2013.1115 PMC4028701

[40] Wurcel AG, Chen G, Zubiago JA, Reyes J, Nowotny KM. Heterogeneity in Jail Nursing Medical Intake Forms: A Content Analysis. J Correct Health Care [Internet]. 2021 Dec [cited 2022 Jul 21];27(4):265–71. Available from: https://www.ncbi.nlm.nih.gov/pmc/articles/PMC8875295/34724807 10.1089/jchc.20.04.0018 PMC8875295

[41] Beckwith CG, Zaller ND, Fu JJS, Montague BTD, Rich JDM. Opportunities to Diagnose, Treat, and Prevent HIV in the Criminal Justice System. Journal of Acquired Immune Defficiency Syndromes. 2010 Dec; 10.1097/QAI.0b013e3181f9c0f7 PMC3017345 21045600

[42] Brinkley-Rubinstein L, Turner WL. Health Impact of Incarceration on HIV-Positive African American Males: A Qualitative Exploration. AIDS Patient Care and STDs [Internet]. 2013 Aug [cited 2022 May 23];27(8):450–8. Available from: https://www.liebertpub.com/doi/10.1089/apc.2012.0457 23968205 10.1089/apc.2012.0457

[43] Iroh PA, Mayo H, Nijhawan AE. The HIV Care Cascade Before, During, and After Incarceration: A Systematic Review and Data Synthesis. American Journal Of Public Health. 2015;105(7):E5–16. 10.2105/AJPH.2015.302635 PMC4463395 25973818

[44] Khan MR, Doherty IA, Schoenbach VJ, Taylor EM, Epperson MW, Adimora AA. Incarceration and high-risk sex partnerships among men in the United States. J Urban Health. 2009 Jul;86(4):584–601. 19459050 10.1007/s11524-009-9348-5 PMC2704271

[45] Knittel AK, Shook-Sa BE, Rudolph J, Edmonds A, Ramirez C, Cohen M, Incarceration and Number of Sexual Partners After Incarceration Among Vulnerable US Women, 2007–2017. Am J Public Health. 2020 Jan;110(S1):S100–8. 31967873 10.2105/AJPH.2019.305410 PMC6987934

[46] Maradiaga JA, Nahvi S, Cunningham CO, Sanchez J, Fox AD. “I Kicked the Hard Way. I Got Incarcerated.” Withdrawal from Methadone During Incarceration and Subsequent Aversion to Medication Assisted Treatments. Journal of Substance Abuse Treatment [Internet]. 2016 Mar 1 [cited 2019 Nov 11];62:49–54. Available from: http://www.sciencedirect.com/science/article/pii/S0740547215002871 26747509 10.1016/j.jsat.2015.11.004 PMC4888768

[47] Rich JD, Beckwith CG, Macmadu A, Marshall BDL, Brinkley-Rubinstein L, Amon JJ, Clinical care of incarcerated people with HIV, viral hepatitis, or tuberculosis. The Lancet [Internet]. 2016 Sep 10 [cited 2022 May 25];388(10049):1103–14. Available from: https://www.sciencedirect.com/science/article/pii/S0140673616303798 10.1016/S0140-6736(16)30379-8 PMC5504684 27427452

[48] Westergaard RP, Kirk GD, Richesson DR, Galai N, Mehta SH. Incarceration Predicts Virologic Failure for HIV-Infected Injection Drug Users Receiving Antiretroviral Therapy. Clinical Infectious Diseases [Internet]. 2011 Oct 1 [cited 2021 Mar 5];53(7):725–31. Available from: 10.1093/cid/cir491 21890777 PMC3202322

[49] Wood E, Li K, Small W, Montaner JS, Schechter MT, Kerr T. Recent Incarceration Independently Associated with Syringe Sharing by Injection Drug Users. Public Health Rep [Internet]. 2005 Mar 1 [cited 2021 Jun 25];120(2):150–6. Available from: 10.1177/003335490512000208 15842116 PMC1497693

[50] Barocas JA, White LF, Wang J, Walley AY, LaRochelle MR, Bernson D, Estimated Prevalence of Opioid Use Disorder in Massachusetts, 2011–2015: A Capture-Recapture Analysis. Am J Public Health. 2018;108(12):1675–81. 30359112 10.2105/AJPH.2018.304673 PMC6236756

[51] Centers for Disease Control and Prevention. CDC National Center for Health Statistics website. 2022 [cited 2022 Jul 19]. Drug Overdose Mortality by State. Available from: https://www.cdc.gov/nchs/pressroom/sosmap/drug_poisoning_mortality/drug_poisoning.htm

[52] Gladden RM, Martinez P, Seth P. Fentanyl Law Enforcement Submissions and Increases in Synthetic Opioid–Involved Overdose Deaths — 27 States, 2013–2014. MMWR Morb Mortal Wkly Rep [Internet]. 2016 [cited 2022 Jul 19];65. Available from: https://www.cdc.gov/mmwr/volumes/65/wr/mm6533a2.htm 10.15585/mmwr.mm6533a2 27560775

[53] O’Donnell JK. Deaths Involving Fentanyl, Fentanyl Analogs, and U-47700 — 10 States, July–December 2016. MMWR Morb Mortal Wkly Rep [Internet]. 2017 [cited 2022 Jul 19];66. Available from: https://www.cdc.gov/mmwr/volumes/66/wr/mm6643e1.htm 10.15585/mmwr.mm6643e1 PMC5689219 29095804

[54] Somerville NJ, O’Donnell J, Gladden RM, Zibbell JE, Green TC, Younkin M, Characteristics of Fentanyl Overdose — Massachusetts, 2014–2016. MMWR Morb Mortal Wkly Rep. 2017 Apr 14;66(14):382–6. 28406883 10.15585/mmwr.mm6614a2 PMC5657806

[55] Cranston K. Notes from the Field: HIV Diagnoses Among Persons Who Inject Drugs — Northeastern Massachusetts, 2015–2018. MMWR Morb Mortal Wkly Rep [Internet]. 2019 [cited 2021 May 7];68. Available from: https://www.cdc.gov/mmwr/volumes/68/wr/mm6810a6.htm 10.15585/mmwr.mm6810a6 PMC6421964 30870405

[56] Madoff L, Brown CM, Lo J, Sánchez S. Joint MDPH and BHPC Clinical Advisory: Increase in newly diagnosed HIV infections among persons who inject drugs in Boston, March 15, 2021 [Internet]. Massachusetts Department of Public Health; 2021 Mar. Available from: https://www.mass.gov/doc/joint-mdph-and-bphc-clinical-advisory-hiv-transmission-through-injection-drug-use-in-boston-march-15-2021

[57] Massachusetts Department of Public Health. Massachusetts HIV/AIDS Epidemiologic Profile, Detailed Data Tables – Data as of 1/1/2020 [Internet]. Massachusetts Department of Public Health, Bureau of Infectious Disease and Laboratory Sciences; 2020 Dec [cited 2021 May 7]. Available from: https://www.mass.gov/lists/hivaids-epidemiologic-profiles

[58] Taylor JL, Ruiz-Mercado G, Sperring H, Bazzi AR. A Collision of Crises: Addressing an HIV Outbreak among People who Inject Drugs in the midst of COVID-19. J Subst Abuse Treat [Internet]. 2021 May [cited 2022 Jul 19];124:108280. Available from: https://www.ncbi.nlm.nih.gov/pmc/articles/PMC8004551/33771280 10.1016/j.jsat.2021.108280 PMC8004551

[59] Heckathorn DD. Respondent-Driven Sampling: A New Approach to the Study of Hidden Populations Studying Hidden Populations. Soc Probs [Internet]. 1997 [cited 2021 Nov 24];44(2):174–99. Available from: https://heinonline.org/HOL/P?h=hein.journals/socprob44&i=184

[60] Lansky A, Abdul-Quader AS, Cribbin M, Hall T, Finlayson TJ, Garfein RS, Developing an HIV Behavioral Surveillance System for Injecting Drug Users: The National HIV Behavioral Surveillance System. Public Health Rep [Internet]. 2007 Jan 1 [cited 2022 Jul 12];122(Supplement 1):48–55. Available from: 10.1177/00333549071220S108 PMC1804107 17354527

[61] Malekinejad M, Johnston LG, Kendall C, Kerr LR, Franco, Sansigolo, Using Respondent-Driven Sampling Methodology for HIV Biological and Behavioral Surveillance in International Settings: A Systematic Review. AIDS and Behavior [Internet]. 2008 Jul [cited 2022 Jul 22];12:105–30. Available from: https://www.proquest.com/docview/211220136/abstract/3EB7EB02D2F343E9PQ/1 10.1007/s10461-008-9421-1 18561018

[62] Centers for Disease Control and Prevention. National HIV Behavioral Surveillance System Round 5: Model Surveillance Protocol [Internet]. 2018 Dec [cited 2022 Jul 12]. Available from: www.cdc.gov/hiv/statistics/systems/nhbs/operations.html

[63] Centers for Disease Control and Prevention. National HIV Behavioral Surveillance System Round 4: Model Surveillance Protocol [Internet]. 2015 Dec [cited 2022 Jul 12]. Available from: www.cdc.gov/hiv/statistics/systems/nhbs/operations.html

[64] Zou G. A Modified Poisson Regression Approach to Prospective Studies with Binary Data. American Journal of Epidemiology [Internet]. 2004 Apr 1 [cited 2022 Jul 25];159(7):702–6. Available from: 10.1093/aje/kwh090 15033648

[65] Zou GY, Donner A. Extension of the modified Poisson regression model to prospective studies with correlated binary data. Statistical Methods in Medical Research [Internet]. 2013 Dec [cited 2022 Jul 25];22(6):661–70. Available from: https://www.proquest.com/docview/1462848429/abstract/50CFC997CCB6409BPQ/1 22072596 10.1177/0962280211427759

[66] Burnett JC, Broz D, Spiller MW, Wejnert C, Paz-Bailey G. HIV Infection and HIV-Associated Behaviors Among Persons Who Inject Drugs — 20 Cities, United States, 2015. MMWR Morb Mortal Wkly Rep [Internet]. 2018 Jan 12 [cited 2021 Jun 16];67(1):23–8. Available from: https://www.ncbi.nlm.nih.gov/pmc/articles/PMC5769798/29324726 10.15585/mmwr.mm6701a5 PMC5769798

[67] Handanagic S, Finlayson T, Burnett JC, Broz D, Wejnert C, National HIV Behavioral Surveillance Study Group. HIV Infection and HIV-Associated Behaviors Among Persons Who Inject Drugs — 23 Metropolitan Statistical Areas, United States, 2018. MMWR Morb Mortal Wkly Rep. 2021 Oct 22;70(42):1459–65. 34673746 10.15585/mmwr.mm7042a1 PMC9361835

[68] Lambdin BH, Kral AH, Comfort M, Lopez AM, Lorvick J. Associations of criminal justice and substance use treatment involvement with HIV/HCV testing and the HIV treatment cascade among people who use drugs in Oakland, California. Addict Sci Clin Pract. 2017 Jun 14;12(1):13. 28610602 10.1186/s13722-017-0078-9 PMC5470222

[69] Farel CE, Golin CE, Ochtera RD, Rosen DL, Margolis M, Powell W, Underutilization of HIV Testing Among Men with Incarceration Histories. AIDS Behav. 2019 Apr;23(4):883–92. 30661215 10.1007/s10461-018-02381-9 PMC9490788

[70] Gwadz M, Cleland CM, Kutnick A, Leonard NR, Ritchie AS, Lynch L, Factors Associated with Recent HIV Testing among Heterosexuals at High Risk for HIV Infection in New York City. Frontiers in Public Health [Internet]. 2016 [cited 2022 Jan 5];4:76. Available from: https://www.frontiersin.org/article/10.3389/fpubh.2016.00076 27200330 10.3389/fpubh.2016.00076 PMC4846660

[71] Wise A, Finlayson T, Sionean C, Paz-Bailey G. Incarceration, HIV Risk–Related Behaviors, and Partner Characteristics Among Heterosexual Men at Increased Risk of HIV Infection, 20 US Cities. Public Health Rep [Internet]. 2019 May 1 [cited 2021 Mar 2];134(1_suppl):63S–70S. Available from: 10.1177/0033354919833435 31059417 PMC6505313

[72] Wise A, Finlayson T, Nerlander L, Sionean C, Paz-Bailey G, NHBS Study Group. Incarceration, Sexual Risk-Related Behaviors, and HIV Infection Among Women at Increased Risk of HIV Infection, 20 United States Cities. J Acquir Immune Defic Syndr. 2017 Jul 1;75 Suppl 3:S261–7. 28604426 10.1097/QAI.0000000000001401

[73] Stone J, Fraser H, Lim AG, Walker JG, Ward Z, MacGregor L, Incarceration history and risk of HIV and hepatitis C virus acquisition among people who inject drugs: a systematic review and meta-analysis. The Lancet Infectious Diseases [Internet]. 2018 Dec 1 [cited 2022 Jul 21];18(12):1397–409. Available from: https://www.thelancet.com/journals/laninf/article/PIIS1473-3099(18)30469-9/fulltext 30385157 10.1016/S1473-3099(18)30469-9 PMC6280039

[74] Ojikutu BO, Srinivasan S, Bogart LM, Subramanian SV, Mayer KH. Mass incarceration and the impact of prison release on HIV diagnoses in the US South. PLOS ONE [Internet]. 2018 Jun 11 [cited 2021 Jun 25];13(6):e0198258. Available from: https://journals.plos.org/plosone/article?id=10.1371/journal.pone.0198258 29889837 10.1371/journal.pone.0198258 PMC5995372

[75] Singer M, Bulled N, Ostrach B, Mendenhall E. Syndemics and the biosocial conception of health. The Lancet [Internet]. 2017 Mar 4 [cited 2022 Jul 21];389(10072):941–50. Available from: https://www.sciencedirect.com/science/article/pii/S014067361730003X 10.1016/S0140-6736(17)30003-X 28271845

[76] Bromberg DJ, Mayer KH, Altice FL. Identifying and Managing Infectious Disease Syndemics in Patients with HIV. Curr Opin HIV AIDS [Internet]. 2020 Jul [cited 2022 Jul 21];15(4):232–42. Available from: https://www.ncbi.nlm.nih.gov/pmc/articles/PMC7376494/32487816 10.1097/COH.0000000000000631 PMC7376494

[77] Hodder SL, Feinberg J, Strathdee SA, Shoptaw S, Altice FL, Ortenzio L, The opioid crisis and HIV in the USA: deadly synergies. The Lancet [Internet]. 2021 Mar 20 [cited 2021 May 24];397(10279):1139–50. Available from: https://www.sciencedirect.com/science/article/pii/S0140673621003913 10.1016/S0140-6736(21)00391-3 33617769

[78] Massachusetts General Laws, Part I, Title XVI, Chapter 111, Section 70F [Internet]. [cited 2024 Feb 27]. Available from: https://malegislature.gov/Laws/GeneralLaws/PartI/TitleXVI/Chapter111/Section70F

[79] Health Resources and Services Administration. Policy Clari cation Notice #16–02 - Ryan White HIV/AIDS Program Services: Eligible Individuals & Allowable Uses of Funds [Internet]. Health Resources and Services Administration Ryan White HIV/AIDS Program; 2018 Oct [cited 2024 Feb 8]. Report No.: PCN #16–02. Available from: https://ryanwhite.hrsa.gov/sites/default/files/ryanwhite/grants/service-category-pcn-16-02-final.pdf

[80] Kavasery R, Maru DSR, Cornman-Homonoff J, Sylla LN, Smith D, Altice FL. Routine opt-out HIV testing strategies in a female jail setting: a prospective controlled trial. PLoS One. 2009 Nov 25;4(11):e7648. 19946370 10.1371/journal.pone.0007648 PMC2777332

[81] de la Flor C, Porsa E, Nijhawan AE. Opt-out HIV and Hepatitis C Testing at the Dallas County Jail: Uptake, Prevalence, and Demographic Characteristics of Testers. Public Health Rep [Internet]. 2017 Nov 1 [cited 2021 Dec 30];132(6):617–21. Available from: 10.1177/0033354917732755 29045799 PMC5692159

[82] Blue C, Buchbinder M, Brown ME, Bradley-Bull S, Rosen DL. Access to HIV care in jails: Perspectives from people living with HIV in North Carolina. PLOS ONE [Internet]. 2022 Jan 24 [cited 2024 Feb 7];17(1):e0262882. Available from: https://journals.plos.org/plosone/article?id=10.1371/journal.pone.0262882 35073350 10.1371/journal.pone.0262882 PMC8786150

[83] Ly W, Cocohoba J, Chyorny A, Halpern J, Auerswald C, Myers J. Perspectives on Integrated HIV and Hepatitis C Virus Testing Among Persons Entering a Northern California Jail: A Pilot Study. J Acquir Immune Defic Syndr. 2018 Jun 1;78(2):214–20. 29474267 10.1097/QAI.0000000000001664

[84] Levano SR, Epting ME, Pluznik JA, Philips V, Riback LR, Zhang C, HIV testing in jails: Comparing strategies to maximize engagement in HIV treatment and prevention. PLoS One. 2023;18(6):e0286805. 37352306 10.1371/journal.pone.0286805 PMC10289455

[85] Alves J, Stewart J, Ruiz-Mercado G, Taylor JL. When Perfect Is the Enemy of Tested: a Call to Scale Rapid HIV Testing for People Who Inject Drugs. J Gen Intern Med. 2022 Aug;37(11):2851–2. 35132547 10.1007/s11606-022-07436-1 PMC8821779

[86] Nijhawan AE, Iroh PA, Porsa E. Acceptability of HIV Testing Among Jail Inmates When Combined With a Blood Test for Tuberculosis. J Correct Health Care [Internet]. 2018 Apr 1 [cited 2021 Mar 2];24(2):120–6. Available from: 10.1177/1078345818762107 29544376 PMC6663493

[87] Faryar KA, Ancona RM, Reau Z, Lyss SB, Braun RS, Rademaker T, HIV detection by an emergency department HIV screening program during a regional outbreak among people who inject drugs. PLOS ONE [Internet]. 2021 May 18 [cited 2023 Dec 7];16(5):e0251756. Available from: https://journals.plos.org/plosone/article?id=10.1371/journal.pone.0251756. 34003855 10.1371/journal.pone.0251756 PMC8130938

[88] Geren KI, Lovecchio F, Knight J, Fromm R, Moore E, Tomlinson C, Identification of acute HIV infection using fourth-generation testing in an opt-out emergency department screening program. Ann Emerg Med. 2014 Nov;64(5):537–46. 24970245 10.1016/j.annemergmed.2014.05.021

[89] Harvey L, Taylor JL, Assoumou SA, Kehoe J, Schechter-Perkins EM, Bernstein E, Sexually Transmitted and Blood-borne Infections Among Patients Presenting to a Low-barrier Substance Use Disorder Medication Clinic. J Addict Med. 2021 Dec 1;15(6):461–7. 34734572 10.1097/ADM.0000000000000801 PMC8569143

